# Molecular Analysis and Risk Factors for *Escherichia coli* Producing Extended-Spectrum β-Lactamase Bloodstream Infection in Hematological Malignancies

**DOI:** 10.1371/journal.pone.0035780

**Published:** 2012-04-23

**Authors:** Patricia Cornejo-Juárez, Carolina Pérez-Jiménez, Jesús Silva-Sánchez, Consuelo Velázquez-Acosta, Fernanda González-Lara, Fernando Reyna-Flores, Alejandro Sánchez-Pérez, Patricia Volkow-Fernández

**Affiliations:** 1 Department of Infectious Diseases, Instituto Nacional de Cancerología (INCan), Secretaría de Salud (Ssa), Mexico City, Mexico; 2 Departament of Epidemiologic Diagnosis, Instituto Nacional de Salud Pública (INSP), Centro de Investigaciones sobre Enfermedades Infecciosas (CISEI), Ssa, Cuernavaca, Morelos, Mexico; Institut de Génétique et Microbiologie, France

## Abstract

**Introduction:**

Patients with hematologic malignancies have greater risk-factors for primary bloodstream infections (BSI).

**Methods:**

From 2004–2009, we analyzed bacteremia caused by extended-spectrum beta-lactamase *Escherichia coli* (ESBL-EC) (*n* = 100) and we compared with bacteremia caused by cephalosporin-susceptible *E. coli* (*n* = 100) in patients with hematologic malignancies.

**Objective:**

To assess the clinical features, risk factors, and outcome of ESBL-EC BSI in patients with hematologic malignancies, and to study the molecular epidemiology of ESBL-EC isolates.

**Results:**

The main diagnosis was acute leukemia in 115 patients (57.5%). Death-related *E. coli* infection was significantly increased with ESBL-EC (34% vs. control group, 19%; *p* = 0.03). Treatment for BSI was considered appropriate in 64 patients with ESBL-EC (mean survival, 245±345 days), and in 45 control patients this was 443±613 (*p* = 0.03). In patients not receiving appropriate antimicrobial treatment, survival was significantly decreased in cases compared with controls (26±122 vs. 276±442; *p* = 0.001). Fifty six of the ESBL-EC isolates were characterized by molecular analysis: 47 (84%) expressed CTX-M-15, two (3.6%) SHV, and seven (12.5%) did not correspond to either of these two ESBL enzymes. No TLA-1 enzyme was detected.

**Conclusions:**

Patients who had been previously hospitalized and who received cephalosporins during the previous month, have an increased risk of ESBL-EC bacteremia. Mortality was significantly increased in patients with ESBL-EC BSI. A polyclonal trend was detected, which reflects non-cross transmission of multiresistant *E.coli* isolates.

## Introduction

Bacterial resistance has increased in recent years, is related with the use of broad-spectrum antibiotics in humans and animals, and is at present considered a public health problem in communities' worldwide [1]. *Enterobacteriaceae* is a frequently found group of microorganisms in hospital-, health-care related, and community-acquired infections [2].

Acquired resistance to β-lactam antibiotics is mainly mediated by extended-spectrum beta-lactamases (ESBLs) [2]. More than 700 distinct types of beta-lactamases have been described [3]. The increasing prevalence of ESBL producers among enterobacterial isolates may be caused by dissemination of resistant traits, proliferation of epidemic strains, or transfer of the resistant gene-carrying plasmids [4]. ESBL-producing *Escherichia coli* (ESBL-EC), particularly those producing CTX-M types, are emerging pathogens worldwide [4].

Knowledge of risk factors for ESBL-EC bacteremia will contribute to identify patients who are at higher risk, in order to start empirical therapy in a more timely fashion with proper coverage against these microorganisms [2]

The objective of this study was to assess the clinical features, risk factors, and outcome of ESBL-EC bloodstream infection (BSI) in patients with hematologic malignancies, and to study the molecular epidemiology of ESBL-EC-carrying isolates in a non-epidemic situation.

## Materials and Methods

### Hospital setting

The Instituto Nacional de Cancerología (INCan) is a tertiary oncology teaching hospital in Mexico, with 150 beds. The hospital has 7,500 hospital discharges, 3,450 surgeries per year and 30,000 chemotherapy-intravenous (IV)-infusion sessions per year.

### Clinical data

We obtained ethical approval from the Instituto Nacional de Cancerología Ethics Committee. We performed a retrospective case-control study in patients with hematologic malignancies, with a positive blood culture for *E. coli*. Cases were considered patients with *E. coli* isolated from blood cultures, resistant to cephalosporins, and inhibition of the resistance with clavulanic acid (ESBL-EC). The control group included patients with a cephalosporin-susceptible *E. coli* positive blood culture, taken ±30 days prior to the date of isolation of the case strain. For patients who underwent multiple hospitalizations during the study period, the earliest *E. coli* isolated from blood culture was considered the index episode. One control patient was selected for each case. We did not obtain informed consent because we included only the strains isolated as part of clinical management and microbiology routine.

The following data were collected: demographic factors; invasive procedures; hospitalizations during the previous 90 days; days of severe neutropenia (<500 cell/mm^3^); chemotherapy treatment and medical procedures (30 days prior to the index infection); presence of severe sepsis or septic shock and use and type of antimicrobial agents employed during the previous month. Bacteremia type was classified in: A) Primary bacteremia; considered in those patients with positive blood cultures who presented clinical sepsis, severe neutropenia (<500 cell/mm^3^) and no specific infection site. B) Secondary bacteremia; when a patient had a specific site of infection with the same strain isolated from the infection site and from blood cultures. C) Catheter related bacteremia; were those patients with long-indwelling catheter and fever or shiver after catheter use, and positive blood culture drawn from the catheter >2 hours of positivity to peripheral positive blood culture, taken on the same day consecutively [5].

Clinical outcome (60 days after the infectious episode) was considered as alive, infection-attributable death, infection-non-related death, and lost to follow-up. Health-care related infection was considered in those patients who had >72 hours of hospitalization or had been hospitalized during the previous 2 weeks [6].

### Bacterial isolates

We included all positive blood cultures taken from January 2004 to December 2009. Blood samples were processed by the automated blood culture BACTEC 9240 System (Becton-Dickinson Microbiology Systems). Appropriate antimicrobial treatment was considered when this had been initiated within the first 48 h of first symptoms and if the patient had received this for at least >72 h, including an antibiotic with susceptibility for the isolated *E. coli* strain.

### Antimicrobial susceptibility testing and detection of ESBL

All *E. coli* isolates were identified by standard microbiological procedures. Detection of antimicrobial susceptibility testing was performed by the automated MicroScan method (Dade-Behring, Sacramento, CA, USA). *E. coli* ATCC 25922 was utilized for quality control accordingly to Clinical and Laboratory Standards Institute (CLSI) guidelines [7].

Identification for ESBL production was performed by the double disk synergism test following CLSI recommendations [7]. *K. pneumonia*e ATCC 700603 was used as positive control for ESBL production.

### Genomic DNA typing

For Pulse-field gel electrophoresis (PFGE) typing, whole cell DNA was obtained according to the method described by Kaufman [8], [9]. DNA was digested with *Xba*I (Gibco, BRL, UK) and separated in a 2% agarose gel (Pulsed Field-Certified, Pronadisa, Madrid, Spain) with a Gene-Path System (Bio-Rad, CHEF MAPER II, USA). Gels were stained with ethidium bromide and analyzed according to the criteria of Tenover et al [10], and Gel Compar II software.

### Plasmid profile

Plasmids were extracted from clinical isolates by the method of Kieser et al [11]. DNA was visualized after vertical electrophoresis in ethidium bromide-stained 0.7% agarose gels. Plasmids R6K (40 kb), RP4 (54 kb), R1 (205 kb), and pUA21 (300 kb) were used as molecular size markers.

### Genetic identification of ESBL

Template DNA was prepared from two fresh colonies resuspended in 100 µl of distilled water and the cells were lysed by heating at 95°C for 10 min. Cellular debris were removed by centrifugation at 15,000 *g* for 2 min; the supernatant was diluted 1∶10 in distilled water and utilized as a source of template DNA for amplification. PCR amplification for each gene was detected using the specific oligonucleotides: *bla*_CTX-M_, CTX-MF, 5′-GCTGTTGTTAGGAAGTGTG-3′ and CTX-MR, 5′-GGTGACGATTTTAGCCGCC-3′; for*bla*_SHV_, those reported previously^1^, and *bla_TLA-1_*, TLA-1F, 5′-TCTCAGCGCAAATCCGCG-3′ and TLA-1R, 5′-CTATTTCCCATCCTTAACTA-3′. PCR amplification was carried out in a 50-µl reaction volume using a thermal cycle 2700 instrument (Applied Biosystems). The reaction mixture contained 5 µl of heat-extracted template DNA, 10 pmol of each primer, 1× reaction buffer, 2 mM MgCl_2_, 0.2 mM of each deoxynucleoside triphosphate, and 1.5 U of *Taq* DNA polymerase. PCR was performed under the following conditions: 5 min of denaturation at 94°C; 25 cycles of 30 sec at 94°C, 30 sec at 58°C, and 30 sec at 72°C, and a final extension for 7 min at 72°C. In all cases, the resulting PCR products were analyzed in 1.5% agarose gels and samples producing one sharp band were purified with a column kit (High Pure™ PCR Purification Kit, Boheringer, USA) and utilized for sequencing reactions with the dideoxy chain termination using an automatic sequencer (ABI PRISM 377-18 kit EL:Taq FS Dye Terminator Cycle Sequencing Fluorescence-Based Sequencing). Primers used for the PCR amplification were employed also for sequencing the amplified PCR products. Amino acid sequences were obtained using the translate tool available at ExPASy (http://www.expasy.ch/tools/dna). Multiple alignments of nucleotide and amino acid sequences were performed with ClustalW (http://clustalw.genome.jp/) software, and the sequences were compared with the *CTX-M-15* (AY044436) and *SHV-1* (AF148850) genes. New genomic data was not generated in this study.

### Statistical methods

Comparison of categorical variables and percentages between groups was carried out by the Pearson chi-square test or the Fisher exact test, as appropriate. Logistic regression analysis was performed to find the association between variables. Reported Odds ratios (ORs) with 95% confidence interval (95% CI) were made. *P* values≤0.05 were considered statistically significant. Variables with a *p* value≤0.1 in the univariate analysis were further tested by means of logistic regression using the forward conditional method. Comparisons of survival were analyzed by means of the log-rank test. Kaplan-Meier curves were performed. Multivariate analysis was performed with logistic regression analysis; all variables statistically significant by univariate analysis were included. The statistical analysis software employed comprised Epi-Info (ver. 6) and STATA (ver. 9.1).

## Results

**Figure 1:**
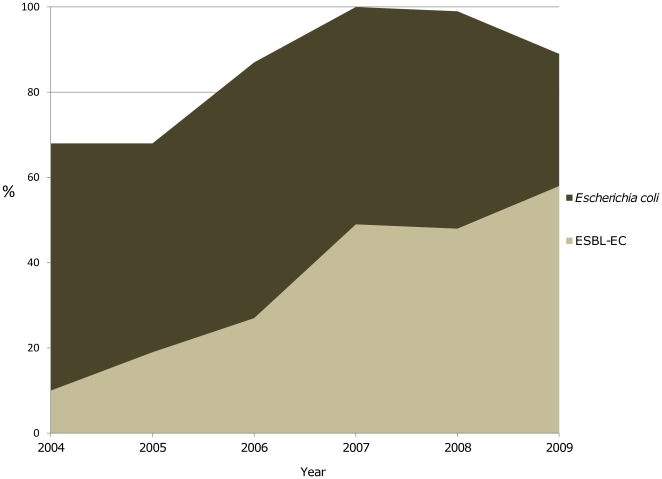
*Escherichia coli*-ESBL isolated from 2004 to 2009. Total number of *Escherichia coli* extended-spectrum beta-lactamases (ESBL) isolated from blood cultures at Instituto Nacional de Cancerología, Mexico City (2004–2009).

A total of 14,764 blood cultures were taken during the study period. Of these, 2,852 (19%) were positive, 670 with *E. coli* (23%) of which 205 episodes (31%) were due to ESBL-EC: 100 patients with hemato-oncologic malignancy were included. A hundred EC- cephalosporin susceptibility patients were included as control group (all also with hemato-oncologic malignancy). There was detected a progressive increase in ESBL-EC isolation from blood cultures from 2004 (14.7%) until 2009 (65.1%) (Figure 1).

Previous hospitalization was present in 93 of ESBL-EC vs. 80 in control (*p* = 0.01). BSI was considered as a health-related infection in 84 cases and in 72 controls (*p* = 0.06). All patients were hospitalized at the hematological ward. Eighteen patients have been bone marrow transplantation, in 6 of them it was performed more than 6 months before the bacteremia. The most frequent underlying condition was diabetes mellitus (n = 14, 7%). Other demographic and clinical characteristics are shown in Table 1 and Table 2.

**Table 1:** Demographic and clinical characteristics in patients with EC-ESBL and controls.

Characteristic	*Escherichia coli*-ESBL (*n* = 100)	Controls (*n* = 100)	Total (*n* = 200)	P
Mean age ± SD* (years)	38.2±17	38.6±17	38.4±17	0.897
Female gender, *n* (%)	55 (55)	42 (42)	97 (49)	0.089
Primary bacteremia	88 (88)	79 (79)	167 (84)	0.226
Secondary bacteremia	10 (10)	17 (17)	27 (14)	
Catheter-related bacteremia	2 (2)	4 (4)	6 (3)	
Previous hospitalizations (within 3 months)	93 (93)	80 (80)	173 (87)	<0.01
Mean days hospitalization	18±12	16±13	17±12	0.01
Mean days of CVC	74±79	67±74	71±77	0.615
Fever and neutropenia	90 (90)	76 (76)	166 (83)	0.01
Mean days of neutropenia	16±27	9±12	13±22	0.02
Previous chemotherapy (within 30 days)	75 (75)	78 (78)	153 (76)	0.711
Septic shock	30 (30)	18 (18)	48 (24)	0.068

* SD: Standard deviation.

**Table 2:** Clinical characteristics in patients with EC-ESBL and controls.

Characteristic		*Escherichia coli*-ESBL (*n* = 100)	Controls (*n* = 100)	Total (*n* = 200)	P
Malignant disease	Leukemia	68 (68)	47 (47)	115 (58)	0.02
	Lymphoma	21 (21)	38 (38)	59 (30)	
	Multiple myeloma	5 (5)	7 (7)	12 (6)	
	Myelodysplasia	6 (6)	8 (8)	14 (7)	
Comorbidity	Diabetes mellitus	6 (6)	8 (8)	14 (7)	0.546
	HIV	3 (3)	0 (0)	3 (1.5)	
	Renal failure	1 81)	3 (3)	4 (2)	
	BMT*	5 (5)	13 (13)	18 (9)	
Chemotherapy	Induction	48	50	98	0.512
	2^nd^ induction	22	19	41	
	3^rd^ or 4^th^ scheme	8	5	13	
	Palliative	12	7	19	
	Conditioning	5	9	14	

* BMT = Bone marrow transplantation.

**Figure 2:**
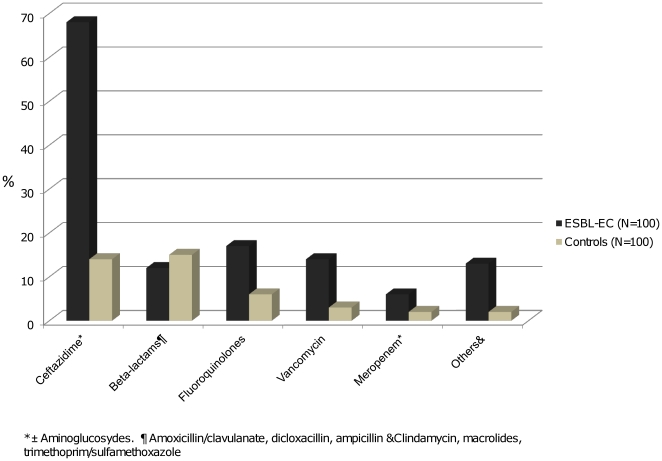
Antimicrobials used in Escherichia coli ESBL and in the control group. Antimicrobials used during the previous month in extended-spectrum beta-lactamase *Escherichia coli* and the *E.coli* control group.

Seventy four patients in the ESBL-EC group and 29 patients in the control group had received antimicrobials during the previous month (*p*<0.0001). The most frequent antibiotics used were cefphalosporins ± aminoglycoside (Figure 2).

All isolates remained fully susceptible to carbapenems. ESBL-EC producers were 95.3% susceptible to amikacin, 42.9% to gentamicin, 5.7% to ciprofloxacin, and 46.7% susceptible to trimethoprim/sulfamethoxazole (TMP/SMX). For *E. coli* control isolates, 100% were susceptible to amikacin (*p* = 0.09), 88% to gentamicin (*p*<0.0001), 60% to ciprofloxacin (*p*<0.0001), and 88% were susceptible to TMP/SMX (*p* = 0.680).

**Figure 3:**
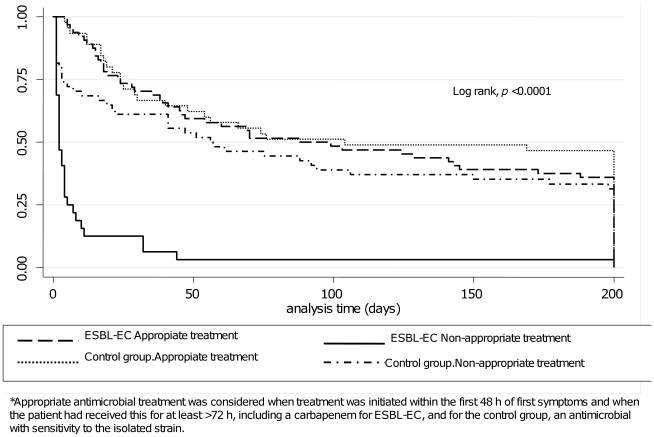
Kaplan Meier survival curve for Escherichia coli ESBL and control patients according to antimicrobial treatment. Kaplan-Meier survival curve for patients with extended-spectrum beta-lactamase *Escherichia coli* and the *E. coli* control group, stratified according to appropriate vs. non-appropriate antimicrobial treatment.

Mean survival in alive patients with ESBL-EC was 390±393 days, while in the control group, this was 812±628 (*p* = 0.009). Treatment for *E. coli* BSI was considered appropriate in 64 patients with ESBL-EC (mean survival, 245±345 days) and in 45 control-group patients (mean survival 443±613 days) (*p* = 0.03). Also, in patients who did not receive an appropriate antimicrobial treatment, mean survival was decreased in ESBL-EC compared with the control group (26±122 vs. 276±442; *p* = 0.001) (Table 3). Analyzing mean survival according to appropriate or non-appropriate antimicrobial treatment in the ESBL-EC group was statistically different (*p* = <0.001). The same analysis in the control group showed no difference (*p* = 0.11). The Kaplan-Meier curve is depicted in Figure 3. There was no relationship between previous antimicrobial use and outcome (*p* = 0.205), neither on comparison of time of neutropenia or mortality (*p* = 0.154).

**Table 3:** Mean survival (days) and outcome according appropriate antimicrobial treatment*.

Outcome of patients	Appropriate antimicrobial treatment				Non-appropriate antimicrobial treatment			
	Total 327±490 (*n* = 109)	Case 245±345 (*n* = 64)	Control 443±613 (*n* = 45)	P = 0.03	Total 177±372 (*n* = 91)	Case 26±122 (*n* = 36)	Control 276±442 (n = 55)	P = 0.001
Alive (*n* = 74)	611±572 (*n* = 51)	411±394 (*n* = 35)	1,050±665 (*n* = 16)	0.0001	577±550 (*n* = 23)	24±29 (*n* = 2)	630±546 (*n* = 21)	0.139
Death- related infection (*n* = 53)	39±78 (*n* = 10)	53±92 (*n* = 7)	7±4 (*n* = 3)	0.423	20±111 (*n* = 43)	30±141 (*n* = 27)	3±5 (*n* = 16)	0.456
Death non-related with infection (*n* = 49)	86±128 (*n* = 33)	37±31 (*n* = 14)	123±159 (*n* = 19)	0.057	86±102 (*n* = 16)	12±17 (*n* = 3)	103±107 (*n* = 13)	0.176
Lost to follow-up (*n* = 24)	82±161 (*n* = 15)	52±76 (*n* = 8)	117±226 (*n* = 7)	0.453	67±125 (*n* = 9)	11±15 (*n* = 4)	12±160 (*n* = 5)	0.256

* Appropriate antimicrobial treatment was considered when this had been initiated within the first 48 h of first symptoms and if the patient had received this for at least >72 h, including an antibiotic with susceptibility for the isolated *E. coli* strain.

In the logistic regression analysis, the variables found to be independently associated with ESBL-EC BSI were: hospitalization during the previous 3 months (OR = 3.6; 95% CI, 1.3–9.9; p = 0.01), and the use of cephalosporins during the previous month (OR = 1.8; 95% CI, 0.9–3.4; P = 0.05).

### Molecular characterization of ESBL-EC producers

**Figure 4:**
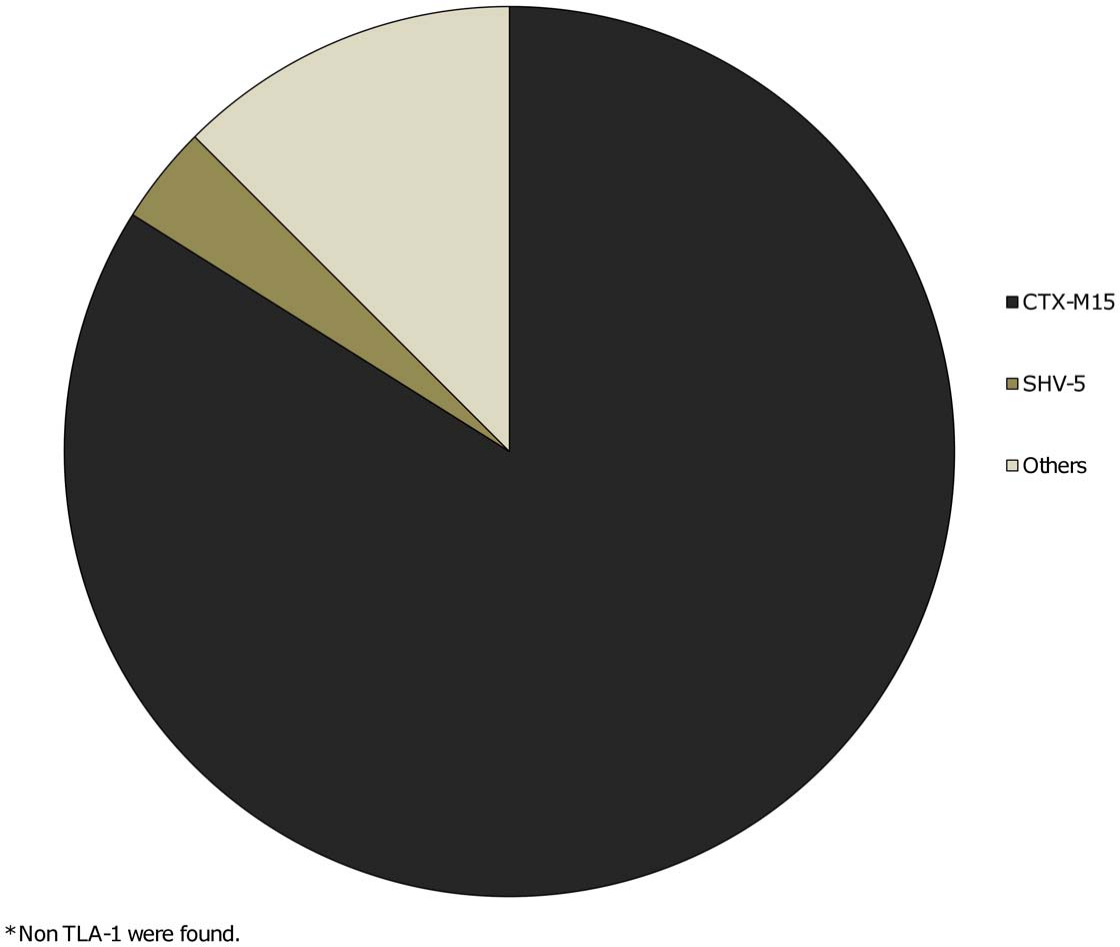
Molecular characterization of Escherichia coli ESBL. Molecular characterization of extended-spectrum beta-lactamase *Escherichia coli* producers (n = 56).

From the 100 EC-ESBL clinical isolates, 56 were randomly selected for further molecular characterization. Macrorestriction genomic DNA analysis showed three minor clonal groups with >85% of similarity; group A (four isolates); group B (three isolates), and C (two isolates); 47 of 56 (83.9%) isolates corresponded to non-genetic-related isolates, suggesting non-cross transmission in this hospital. A positive PCR product of the expected size of 810 pb kb that corresponded to the *bla-*_CTX-M_ ESBL was identified in 84% (47/56). Figure 4. Concerning the plasmids contained in ESBL-EC producers, the majority of the isolates analyzed harbored from 1–3 different plasmids (size range, 60–275 kb).

## Discussion

Over the past few years, a significant increase in the number of ESBL-EC-associated BSI has being reported in several parts of the world [2], [3]. *E. coli* is the most frequent microorganism isolated from blood cultures in our hospital; it represents 20% of all of the isolates from BSI, and of these, 37.8% are ESBLs, although the proportion of ESBLs isolated has increase steadily every year since its first isolation in 2004 representing 64% of the is *E coli* isolates from BSI during 2009. The incidence of ESBL-EC in other medical centers is reported as 4–6% in Latin America (1997–2000), 5% in the Asian Pacific and South Africa (1997–1999), 5% in Europe (1997–1999), and 1.5–16.7% in Taiwan (2002–2006) [12]–[15]. There is a trend toward increasing ESBL-EC incidence with substitution of susceptible strains (Figure 1).

Genotyping of ESBL-EC isolates indicated in the majority of the isolates non-relatedness, suggesting non-cross transmission; this situation has been reported previously by other authors [12]. In Mexico, the most frequent enterobacterial-ESBL producers causing hospital acquired infections has been identified the SHV-5 enzyme [15]–[17] and more recently, it has been substituted by CTX-M-15 ESBL, a phenomenon described already in other parts in the world [16]–[19]. In this study we detected non-genetic-related groups among ESBL-EC-produced clinical isolates, while the CTX-M-15 enzyme was prevalent; this situation suggests plasmid-mediated dissemination of the *bla-_CTX-M-15_* gene, as was identified with the *bla-_SHV-5_* gene. CTX-M-producing bacteria acquire resistance to all generations of cephalosporins, while remaining susceptible to carbapanems [20], as we could see in this study.

In cancer patients with prolong hospitalization and neutropenia who had received antibiotics for prolonged time periods are factors that enhance the opportunity for these bacteria to cause infections [21]–[23]. In this institution we do not use antimicrobial prophylaxis in neutropenic patients. We found that the previous cephalosporins use, was statistically significantly higher in ESBL-EC harboring patients than in controls. It is important to consider that the group of leukemia includes more patients with ESBL-EC BSI, it must be related with a more aggressive chemotherapy and a deeper and longer neutropenia stage compared with control patients.

A determining factor in the outcome of infected patients is choosing the appropriate empirical therapy within the first 48 h of the first symptoms and within the first 24 h of the positive blood culture. The majority of authors have found that inadequate antimicrobial therapy is associated with poorer outcome [24]–[26].

Carbapenems use has been associated with the low risk of death in cases of serious infections caused by these pathogens [25], [26]; all of our isolates remained fully susceptible to carbapenems *in vitro*; thus, these antimicrobials are first line therapy in patients suspected of having ESBLs.

The presence of ESBL has been associated with increased mortality, longer duration of hospitalization and increased hospital costs [1], [24]–[27]. Previous studies have found that malignancy is a mortality-associated risk factor, and patient with a ESBL-EC isolated in a blood-culture have ∼4 times greater overall mortality compared with non-ESBL-producing isolates [28], [29]. Crude mortality rates for bacteremia in neutropenic patients range from 12 to 42%; with the highest rates usually related with gram-negative bacteria, and reached 83% among those hospitalized in an intensive care unit [15]. In an eight year survey, the overall mortality associated with BSI caused by *E.coli* in patients with hematological malignancies was 20.9% [15]. In this study, mortality was increased in patients with ESBL-EC BSI vs. control patients (34 vs. 19%; *p* = 0.03), the sole isolation of an ESBL-EC was associated with an increase risk of death.

The results herein reported may not be applicable in settings with a different epidemiological context; however, as control and case patients were selected from the same hospital and same clinical ward, the potential of selection bias should be negligible.

The prevalence of ESBL-EC gastrointestinal colonization among patients with neutropenic cancer has been reported to be around 30% in other Spanish hospitals in Spain [30], we have no knowledge of it in our hospital [30], [31]. It is absolutely necessary to proceed to a monitoring of fecal-colonization in these patients and develop anti-infective therapeutic protocols tailored to the results of this colonization.

Patients with hemato-oncological malignancies, particularly patients with leukemia, who have been hospitalized for a long period and who have received cephalosporins during the previous month have a major risk of ESBL-EC bacteremia. Mortality is increased in ESBL-EC BSI, even in patients who received appropriate antimicrobial treatment. Multiresistant strains reflect a polyclonal trend that is substituing susceptible isolates over time.

## References

[1] Peña C, Gudiol C, Calatayud L, Tubau F, Dominguez MA, Pujol M (2008). Infections due to Escherichia coli producing extended-spectrum beta-lactamase among hospitalized patients: factors influencing mortality.. J Hosp Infect.

[2] Gudiol C, Calatayud L, García-Vidal C, Cisnal M, Sanchez-Ortega I (2010). Bacteraemia due to extended-spectrum beta-lactamase-producing Escherichia coli (ESBL-EC) in cancer patients: clinical features, risk factors, molecular epidemiology and outcome.. J Antimicrob Chemother.

[3] Garza-González GE, Mendoza Ibarra SI, Llaca-Díaz JM, González GM (2011). Molecular characterization and antimicrobial susceptibility of extended-spectrum beta-lactamase-producing Enterobacteriaceae isolates at a tertiary-care centre in Monterrey, Mexico.. J Med Microbiol.

[4] Trecarichi EM, Tumbarello M, Spanu T, Caira M, Fianchi L (2009). Incidence and clinical impact of extended-spectrum-beta-lactamase (ESBL) production and fluoroquinolone resistance in bloodstream infections caused by Escherichia coli in patients with hematological malignancies.. J Infect.

[5] Blot F, Nitenberg G, Brun-Buisson C (2000). New tools in diagnosing catheter-related infections.. Support Care Cancer.

[6] Garner JS, Jarvis WR, Emori TG, Horan TC, Hughes JM (1988). CDC definitions for nosocomial infections.. Am J Infect Control.

[7] Clinical and Laboratory Standards Institute (2006). Performance Standards for Antimicrobial Susceptibility Testing..

[8] Kaufmann ME (1988). Pulse-field gel electrophoresis.. Methods Mol Med.

[9] Akpaka PE, Legall B, Padman J (2010). Molecular detection and epidemiology of extended-spectrum beta-lactamase genes prevalent in clinical isolates of Klebsiella pneumoniae and E. coli from Trinidad and Tobago.. West Indian Med J.

[10] Tenover FC, Arbeit RD, Goering RV, Mickelsen PA, Murray BE (1995). Interpreting chromosomal DNA restriction patterns produced by pulsed-field gel electrophoresis: criteria for bacterial strain typing.. J Clin Microbiol.

[11] Kieser T (1984). Factors affecting the isolation of CCC DNA from Streptomyces lividans and Escherichia coli.. Plasmid.

[12] Pérez F, Endimiani A, Hujer KM, Bonoma RA (2007). The continuing challenge of ESBLS.. Curr Opin Pharmacol.

[13] Coque TM, Barquero F, Cantón R (2008). Increasing prevalence of ESBL-producing Enterobacteriaceae in Europe. (Abstract).. Euro Surveill.

[14] Paterson DL, Bonomo RA (2005). Extended spectrum beta-lactamases: a clinical update.. Clin Microbiol Rev.

[15] Yu WL, Chuang YCH, Rasmussen JW (2006). Extended-spectrum beta-lactamases in Taiwan: epidemiology, detection, treatment and infection control.. J Microbiol Immunol Infect.

[16] Miranda G, Castro N, Leaños B, Valenzuela A, Garcia-Ramos U (2004). Clonal and horizontal dissemination of Klebsiella pneumoniae expressing SHV-5 extended-spectrum beta-lactamase in a Mexican pediatric hospital.. J ClinMicrobiol.

[17] Silva J, Gatica R, Aguilar C, Becerra Z, Garza-Ramos U (2001). Outbreak of infection with extended-spectrum beta-lactamase-producing Klebsiella pneumoniae in a Mexican hospital.. J Clin Microbiol.

[18] Mosqueda-Gómez JL, Montano-Loza A, Rolon AL, Cervantes C, Bobadilla-del-Valle JM (2008). Molecular epidemiology and risk factors of bloodstream infections caused by extended-spectrum beta-lactamase-producing Klebsiella pneumoniae. A case-control study.. Int J Infect Dis.

[19] Silva-Sánchez J, Garza-Ramos JU, Reyna-Flores F, Sanchez-Perez A, Rojas-Moreno T (2011). Extended-spectrum beta-lactamase-producing Enterobacteriaceae causing nosocomial infections in Mexico. A retrospective and multicenter study.. Arch Med Res.

[20] Chong Y, Yakushiji H, Ito Y, Kamimura T (2010). Cefepime-resistant Gram-negative bacteremia in febrile neutropenic patients with hematological malignancies.. Int J Infect Dis.

[21] Rodríguez-Baño J, Navarro MD, Romero L, Muniain MA, Cueto M (2008). Risk-factors for emerging bloodstream infections caused by extended-spectrum beta-lactamase-producing Escherichia coli.. Clin Microbiol Infect.

[22] Ramphal R, Ambrose PG (2006). Extended-spectrum β-lactamases and clinical outcomes: current data.. Clin Infect Dis.

[23] Wu UI, Wang JL, Chen WC, Chang SC, Chen YC (2011). Risk factors and outcomes of Escherichia coli bacteremia caused by strains that produce CTX-M or non-CTX-M extended-spectrum-beta-lactamases.. Eur J Clin Microbiol Infect Dis.

[24] Hu B, Ye H, Xu Y, Ni Y, Hu Y (2010). Clinical and economic outcomes associated with community-acquired intra-abdominal infections caused by extended spectrum beta-lactamase (ESBL) producing bacteria in China.. Curr Med Res Opin.

[25] Hyle EP, Lipworth AD, Zaoutis TE, Nachamkin I, Bilker WB (2005). Impact of inadequate initial antimicrobial therapy on mortality in infections due to extended-spectrum beta-lactamase-producing Enterobacteriaceae.. Arch Inter Med.

[26] Peña C, Gudiol C, Tubau F, Sabalis M, Pujol M (2006). Risk-factors for acquisition of extended-spectrum beta-lactamase-producing Escherichia coli among hospitalized patients.. Clin Microbiol Infect.

[27] Lautenbach E, Patel JB, Bilker WB, Edelstein PH, Fishman NO (2001). Extended-spectrum beta-lactamase-producing Escherichia coli and Klebsiella pneumoniae: risk factors for infection and impact of resistance on outcomes.. Clin Infect Dis.

[28] Chen CY, Tsay W, Tang JL, Tien HF, Chen YC (2009). Epidemiology of bloodstream infections in patients with haematological malignancies with and without neutropenia.. Epidemiol Infect.

[29] Wang FD, Lin ML, Liu CY (2005). Bacteremia in patients with hematological malignancies.. Chemotherapy.

[30] Valverde A, Coque TM, Sánchez-Moreno MP, Rollán A, Baquero F (2004). Dramatic increase in prevalence of fecal carriage of extended-spectrum beta-lactamase-producing Enterobacteriaceae during nonoutbreak situations in Spain.. J Clin Microbiol.

[31] Garza-Ramos U, Martinez-Romero E, Silva-Sanchez J (2007). SHV-type extended-spectrum beta-lactamase (ESBL) are encoded in related plasmids from enterobacteria clinical isolates from Mexico.. Salud Publica Mex.

